# Supplementary material for: Identification of photoperiod-induced specific miRNAs in the adrenal glands of Sunite sheep (Ovis aries)
Source: Front Vet Sci. 2022 Jul 22;9:888207. doi: 10.3389/fvets.2022.888207 (PMC9354845; doi:10.3389/fvets.2022.888207)
Supplement: Supplementary file 1 [file Data_Sheet_1.ZIP › Supplementary file/miRDeep2 file.zip!/miRDeep2 file/Novel_Structure/LP42A2/Novel_590.pdf]

| | | |
|---|---|---|
| Provisional ID | : | NC_019465.2_16674 |
| Score total | : | 1.5 |
| Score for star read(s) | : | -1.3 |
| Score for read counts | : | 0 |
| Score for mfe | : | 1.2 |
| Score for randfold | : | 1.6 |
| Score for cons. seed | : | |
| Total read count | : | 100 |
| Mature read count | : | 100 |
| Loop read count | : | 0 |
| Star read count | : | 0 |

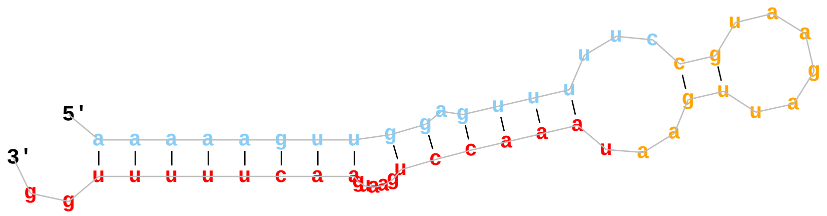


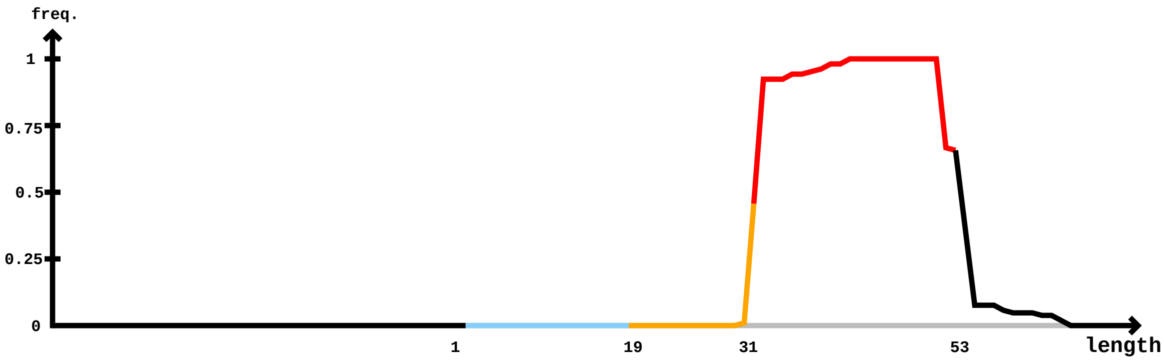


Star Mature

```
5'- aaugcauauauauuaauaaaauguguauuuacaggguuggccaaaaaguuggaguuuuuccguaagauugaauaaaccugaaugaacuuuuuggccaacccauuaauauuau   -3'    exp
    (((((((((..........)))))))))....(((((((((((((((((((.((((...((......))...)))))).....))))))))))))))))).........    reads    mm    sample
    ......................................................................auaaaccugaaugaacuuuuu....................    1    0    seq
    .......................................................................Gaaaccugaaugaacuuuuu....................    1    1    seq
    .......................................................................Caaaccugaaugaacuuuuu....................    3    1    seq
    .......................................................................uaaaccugaaugaacuuuuug...................    1    0    seq
    .......................................................................Aaaaccugaaugaacuuuuugg..................    42    1    seq
    ........................................................................aaaccCgaaugaacuuuuu....................    28    1    seq
    ........................................................................aaaccugaaugaacuuuuu....................    2    0    seq
    ........................................................................aaaccugaaugaacuuuuugg..................    5    0    seq
    ........................................................................aaaccugaaugaacuuuuugA..................    3    1    seq
    ........................................................................aaaccugaaugaacuuuuugU..................    4    1    seq
    ........................................................................aaGccugaaugaacuuuuuggc.................    1    1    seq
    ........................................................................aaaccCgaaugaacuuuuuggc.................    6    1    seq
    ...........................................................................ccugaaugaacuuuuuggccaG..............    1    1    seq
    ...........................................................................ccugaaugaacuuuuuggccaU..............    1    1    seq
    .............................................................................uAaaugaacuuuuuggccaac.............    1    1    seq
    ..............................................................................Aaaugaacuuuuuggccaacccauua.......    1    1    seq
    ...............................................................................aaugaacuuuuuggccaaccca..........    1    0    seq
    ...............................................................................aaugaacuuuuuggccaacccauu........    1    0    seq
    .................................................................................ugaacuuuuuggccaacccauu........    1    0    seq
    .................................................................................ugaacuuuuuggccaacccauua.......    1    0    seq
```
